# 3D-Printed PMMA-Regulated PAN-Based Gel Polymer Electrolytes for Lithium Metal Batteries

**DOI:** 10.3390/molecules31173017

**Published:** 2026-08-28

**Authors:** Jiajia Dong, Xinghua Liang, Yangying Ou, Qinglie Mo, Pengzhen Chen, Lei Zhang, Lingxiao Lan

**Affiliations:** Guangxi Key Laboratory of Automobile Components and Vehicle Technology, Guangxi University of Science and Technology, Liuzhou 545006, China; 16650298814@163.com (J.D.); lxh304@126.com (X.L.); 15697920119@163.com (Y.O.); 100001683@gxust.edu.cn (Q.M.); 15110761348@163.com (P.C.); zzz593756895@163.com (L.Z.)

**Keywords:** gel polymer electrolytes, 3D printing, PAN/PMMA, lithium-ion batteries

## Abstract

Gel polymer electrolytes (GPEs) have emerged as promising electrolytes for lithium metal batteries owing to their high ionic conductivity, mechanical flexibility, and reduced risk of electrolyte leakage. However, PAN-based GPEs still suffer from limited ion transport caused by the semi-crystalline structure of PAN chains. In this work, polyacrylonitrile (PAN)/poly(methyl methacrylate) (PMMA)/lithium aluminum titanium phosphate (LATP)/lithium bis(trifluoromethanesulfonyl)imide (LiTFSI) gel polymer electrolytes were fabricated via direct ink writing (DIW) 3D printing, where PMMA was introduced to regulate the PAN matrix and enhance Li^+^ transport. The results reveal that PMMA incorporation effectively reduces PAN crystallinity, increases the amorphous fraction, and modifies the local functional-group environment of the polymer matrix, while LATP fillers further improve ionic transport and mechanical stability. The optimized PPM8:2 gel polymer electrolyte delivers a room-temperature ionic conductivity of 4.22 × 10^−4^ S cm^−1^, a Li^+^ transference number of 0.624, and an electrochemical stability window of 4.75 V. When applied in LiFePO_4_|Li batteries, it maintains a discharge capacity of approximately 150 mAh g^−1^ after 100 cycles at 0.1 C with excellent rate capability and cycling stability. This work provides an effective approach to developing PAN-based gel polymer electrolytes for high-performance lithium metal batteries.

## 1. Introduction

With the rapid development of electric vehicles, portable electronic devices, and large-scale energy storage systems, the demand for energy storage devices with high energy density and enhanced safety has been continuously increasing [1,2,3]. Conventional lithium-ion batteries widely employ flammable organic liquid electrolytes, which exhibit high ionic conductivity and excellent electrode wettability [4,5,6]. However, the risks associated with electrolyte leakage, poor thermal stability, and the inability to effectively suppress lithium dendrite formation severely hinder the further development of high-energy-density lithium batteries [7]. Therefore, developing advanced electrolyte systems with improved safety and electrochemical stability has become essential for next-generation lithium battery technologies.

Among various emerging electrolyte systems, gel polymer electrolytes (GPEs) have attracted extensive attention due to their ability to immobilize liquid electrolytes within a polymer framework while simultaneously combining the high ionic conductivity of liquid electrolytes with the mechanical flexibility, excellent interfacial contact, and good processability of polymer materials [8,9]. Compared with conventional liquid electrolytes, GPEs can effectively reduce electrolyte leakage and improve battery safety [10]. Meanwhile, compared with inorganic ceramic solid electrolytes, their flexible polymer networks can alleviate solid–solid interfacial contact issues and reduce interfacial resistance [11]. However, most reported GPEs still suffer from insufficient mechanical strength, limited ionic transport capability [12], and unstable electrode/electrolyte interfaces during long-term cycling [13]. Therefore, developing GPEs with simultaneously enhanced ionic conductivity, mechanical robustness, and interfacial stability remains highly desirable.

Polyacrylonitrile (PAN) has been regarded as a promising polymer matrix for GPEs owing to its high dielectric constant, wide electrochemical stability window, and polar nitrile groups (−C≡N) that promote lithium salt dissociation [14,15]. Ren [16] fabricated a hybrid gel polymer electrolyte composed of PAN, LiTFSI, and lithium aluminum titanium phosphate (LATP), demonstrating that the incorporation of LATP could improve the mechanical stability of the electrolyte and establish fast Li^+^ transport pathways, thereby enhancing electrochemical performance. However, PAN is a typical semi-crystalline polymer, and its highly ordered chain arrangement restricts polymer segmental motion, resulting in limited ion transport efficiency [17]. To address these limitations, various modification strategies have been explored for PAN-based GPEs. Yao [18] reported that the introduction of EC and PC plasticizers significantly improved the ionic transport properties of PAN-based polymer electrolytes but simultaneously weakened their mechanical stability. Lee [19] developed PAN/ceramic composite electrolyte membranes and demonstrated that the incorporation of Al_2_O_3_ ceramic fillers could enhance thermal stability and mechanical strength while promoting ion transport. Nevertheless, because inert ceramic fillers cannot directly participate in Li^+^ migration, their ability to enhance ionic conductivity remains limited [20]. Therefore, designing PAN-based GPE systems that can simultaneously enhance mechanical properties and ionic transport capability remains a critical challenge. Poly(methyl methacrylate) (PMMA) is a representative amorphous polymer featuring excellent film-forming ability, high mechanical strength, and good chemical stability [21]. The carbonyl groups (C=O) in PMMA chains can coordinate with Li^+^ ions and participate in lithium-ion transport processes [22]. Moreover, its amorphous structure provides increased free volume and facilitates polymer segmental motion, thereby improving ionic conductivity [23]. Therefore, integrating the excellent electrochemical stability and lithium salt dissociation ability of PAN with the amorphous characteristics of PMMA is expected to achieve synergistic optimization of polymer framework stability and ion-transport behavior in GPEs.

Recently, the incorporation of highly conductive ceramic fillers into gel polymer electrolytes (GPEs) has been regarded as an effective strategy for further improving their overall performance [24]. Active ceramic fillers can not only enhance the mechanical robustness and thermal stability of gel polymer networks but also regulate polymer chain arrangement, reduce polymer crystallinity [25,26], and provide additional Li^+^ transport pathways, thereby promoting ionic migration within the gel matrix [27]. Among various ceramic fillers, NASICON-type Li_1+x_Al_x_Ti_2−x_(PO_4_)_3_ (LATP) has attracted considerable attention in gel polymer electrolyte systems due to its high room-temperature ionic conductivity in the range of 10^−4^–10^−3^ S cm^−1^, excellent thermal stability, and favorable chemical compatibility [28,29]. Liu [30] demonstrated that the incorporation of LATP could effectively regulate the polymer matrix structure, reduce polymer crystallinity, and facilitate rapid Li^+^ migration within the gel network, resulting in improved ionic transport capability and cycling stability of electrolytes. Therefore, introducing LATP into PAN-based gel polymer electrolyte systems is expected to further optimize the gel network structure and achieve synergistic enhancement of ionic conductivity, mechanical properties, and interfacial stability.

The fabrication strategy of gel polymer electrolyte membranes also plays a crucial role in determining their structural uniformity and electrochemical performance [31]. Currently, gel polymer electrolytes are mainly fabricated through conventional methods such as solution casting [32], tape casting [33], and hot pressing [34]. However, these approaches may face limitations in precise thickness control, reproducible structural patterning, and the fabrication of customized or complex architectures, which restrict their flexibility for rational electrolyte structure design. Recently, additive manufacturing technologies have been increasingly applied for the precise fabrication of battery electrolyte membranes [35]. In contrast, direct ink writing (DIW) 3D printing enables programmable layer-by-layer deposition, high material utilization, and flexible control over membrane geometry and deposition patterns, providing a versatile route for fabricating structurally tailored electrolyte membranes [36]. Ping [37] successfully fabricated LLZTO-, LATP-, and LLTO-based solid electrolyte films through a printing-assisted rapid sintering strategy and achieved layer-by-layer fabrication and stable cycling performance of printed all-solid-state batteries, demonstrating the potential of additive manufacturing technologies for advanced battery fabrication. However, research on DIW-printed gel polymer electrolytes remains relatively limited, particularly for PAN-based gel polymer electrolyte systems, where the regulation of polymer structures and the construction of efficient Li^+^ transport networks through printing technology have rarely been explored.

Based on these considerations, this work employs DIW 3D printing technology to fabricate PAN/PMMA/LATP/LiTFSI gel polymer electrolytes. By integrating the excellent electrochemical stability and ability of PAN to promote lithium salt dissociation, the amorphous structural characteristics of PMMA, and the fast lithium-ion transport capability of LATP, the polymer segmental mobility, mechanical stability, and ionic transport properties are simultaneously optimized. The effects of PAN/PMMA composition regulation on the microstructure, thermal stability, ion transport behavior, and electrochemical performance of the gel polymer electrolytes are systematically investigated. This work provides an effective strategy for the design and fabrication of high-performance printable gel polymer electrolytes.

## 2. Results and Discussion

To systematically investigate the effect of PMMA incorporation on the structure and electrochemical properties of PAN-based gel polymer electrolytes, a series of PAN/PMMA/LiTFSI/LATP electrolytes with different PAN/PMMA mass ratios were prepared. The samples were denoted as PPM9:1, PPM8:2, PPM7:3, PPM6:4, and PPM5:5, corresponding to PAN/PMMA mass ratios of 9:1, 8:2, 7:3, 6:4, and 5:5, respectively. A PAN/LiTFSI/LATP electrolyte without PMMA was used as the control and is denoted as PAN throughout the manuscript. For all electrolyte samples, the contents of LiTFSI and LATP were fixed at 30 wt% and 10 wt%, respectively, relative to the total polymer mass. Unless otherwise specified, the sample designations and compositions described above are used throughout the following discussion.

### 2.1. Structural Evolution and Physicochemical Characteristics

The incorporation of PMMA is expected to regulate the semi-crystalline structure of PAN and modify the local chemical environment of the polymer matrix, thereby affecting polymer segmental motion and Li^+^ transport. Therefore, X-ray diffraction (XRD), Fourier transform infrared spectroscopy (FTIR), thermogravimetric analysis (TGA), and differential scanning calorimetry (DSC) were first employed to elucidate the structural evolution, molecular interactions, and thermal characteristics of the PAN/PMMA-based gel polymer electrolytes.

**Figure 1:**
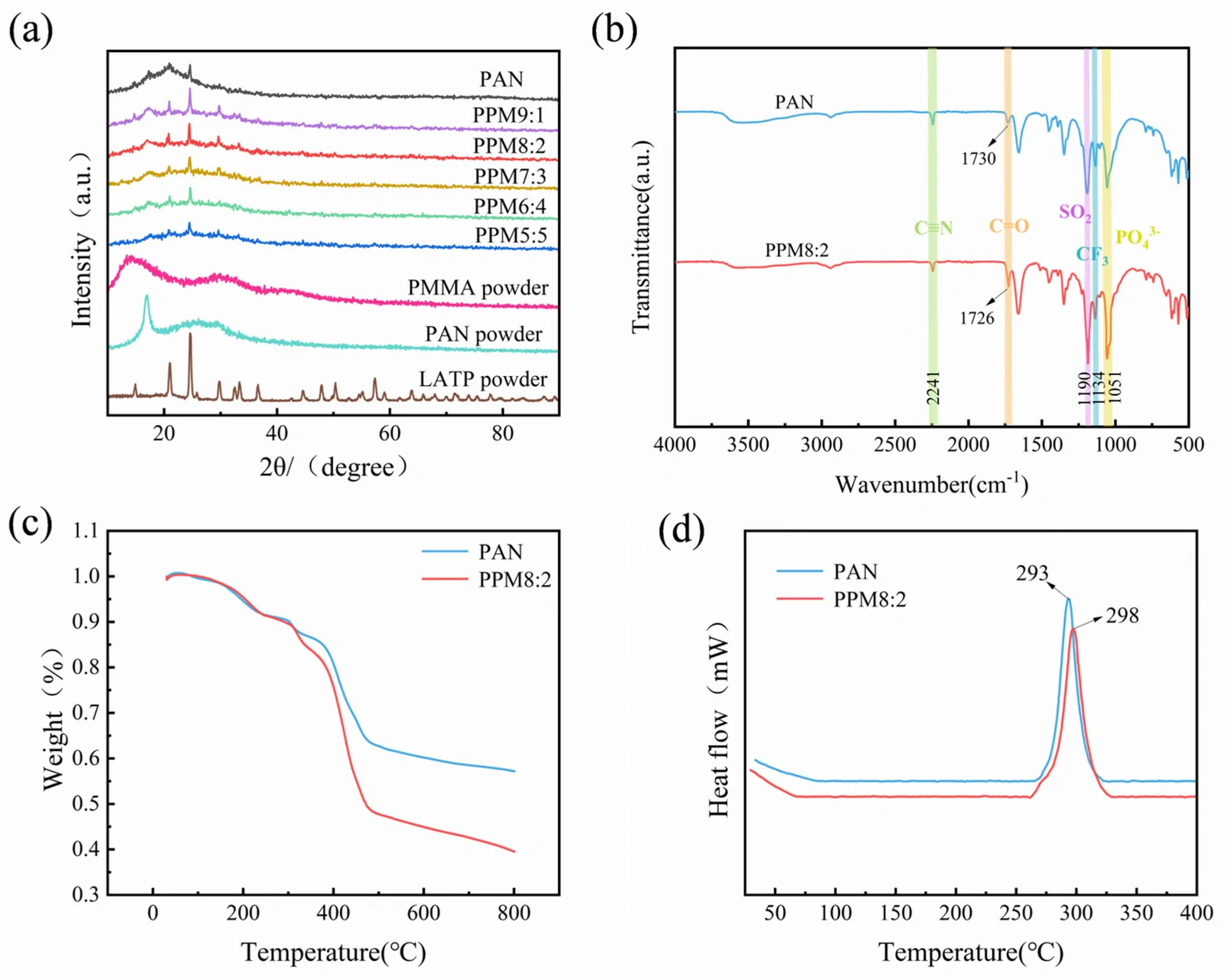
(**a**) X-ray diffraction (XRD) patterns of PAN, PMMA, and LATP powders, the PAN-based electrolyte, and PAN/PMMA-based gel polymer electrolytes with different PAN/PMMA mass ratios; (**b**) Fourier transform infrared (FTIR) spectra of the PAN-based electrolyte and PPM8:2 gel polymer electrolyte; (**c**) thermogravimetric analysis (TGA) curves of the PAN-based electrolyte and PPM8:2 gel polymer electrolyte; (**d**) differential scanning calorimetry (DSC) curves of the PAN-based electrolyte and PPM8:2 gel polymer electrolyte.

Figure 1a presents the XRD patterns of LATP, PAN, and PMMA powders, the PAN-based electrolyte, and PAN/PMMA-based gel polymer electrolytes with different PAN/PMMA mass ratios. The pristine LATP powder exhibits sharp diffraction peaks, indicating its highly crystalline structure [38]. Pure PAN shows broad diffraction peaks at approximately 2θ = 17° and 29°, corresponding to its semi-crystalline polymer chains [39], whereas PMMA displays only a broad diffuse halo, confirming its amorphous nature [40]. For the PAN-based gel polymer electrolyte (PAN/LiTFSI/LATP), the characteristic diffraction peaks of LATP remain unchanged, while a broad peak around 17° associated with PAN semicrystalline domains is still observed, suggesting partial chain ordering within the PAN matrix. With increasing PMMA content, this peak gradually weakens and broadens, whereas the LATP diffraction peaks remain nearly unchanged. These results indicate that PMMA incorporation does not affect the LATP crystal structure but effectively suppresses the ordered packing of PAN chains, increasing the amorphous fraction of the gel polymer network. The enlarged amorphous regions facilitate polymer segmental motion and provide favorable environments for Li^+^ transport [41].

To investigate the local chemical environment of the gel polymer electrolytes, FTIR spectra of the PAN-based electrolyte and PPM8:2 were analyzed, as shown in Figure 1b. Both samples exhibit the characteristic C≡N stretching vibration of PAN at approximately 2241 cm^−1^, with no appreciable shift after PMMA incorporation. A weak absorption feature is observed around 1730 cm^−1^ in the PAN-based electrolyte. Since PAN does not intrinsically contain carbonyl groups, this weak feature may be associated with trace carbonyl-containing species arising from material composition or processing [42,43]. After PMMA incorporation, PPM8:2 exhibits a stronger carbonyl-region absorption band at approximately 1726 cm^−1^, which is consistent with the characteristic ester C=O stretching vibration of PMMA [44]. The enhanced carbonyl-related absorption supports the successful incorporation of PMMA and indicates changes in the carbonyl-related vibration environment after PMMA incorporation. In addition, the bands at approximately 1134 and 1190 cm^−1^ are assigned to the CF_3_ and SO_2_ vibrations of LiTFSI [45], respectively, while the band near 1051 cm^−1^ corresponds to the PO_4_^3−^ framework vibration of LATP [46]. Overall, the FTIR results confirm the presence of the constituent components and support the successful incorporation of PMMA into the PAN-based gel polymer electrolyte matrix.

After establishing the structural and molecular characteristics of the electrolytes, their thermal stability was evaluated by TGA. Figure 1c presents the thermogravimetric analysis (TGA) curves of the PAN-based electrolyte and PPM8:2 gel polymer electrolyte. No obvious weight loss is observed below 200 °C for both samples, indicating the excellent thermal stability of the resulting gel polymer networks. Upon further heating, both gel polymer electrolytes undergo continuous thermal decomposition processes. The weight loss within 300–500 °C is mainly attributed to the thermal decomposition of polymer components, while the further weight decrease at higher temperatures corresponds to the decomposition of organic chains. Compared with the PAN gel polymer electrolyte, the PPM8:2 sample exhibits a more pronounced weight loss around 400 °C and slightly lower residual mass, which is mainly associated with the thermal decomposition of PMMA at elevated temperatures. Nevertheless, the PPM8:2 gel polymer electrolyte maintains sufficient thermal stability within the practical operating temperature range of batteries, indicating that PMMA incorporation does not substantially compromise the thermal stability of the gel system.

To further investigate the influence of PMMA on the thermal transition behavior of the PAN-based gel network, differential scanning calorimetry (DSC) measurements were performed, as shown in Figure 1d. The PAN-based electrolyte exhibits a pronounced exothermic peak at approximately 293 °C, which is associated with the cyclization reaction of PAN chains. After PMMA incorporation, the exothermic peak of the PPM8:2 sample shifts slightly to around 298 °C and shows an altered peak profile, indicating that PMMA incorporation affects the thermal reaction behavior of the PAN-based polymer matrix. This change may be associated with the regulation of PAN chain arrangement and the modified local polymer environment induced by PMMA incorporation. These results suggest that PMMA alters the thermal transition characteristics of the PAN-based gel network without substantially compromising its thermal stability.

### 2.2. Morphology and Mechanical Properties

The incorporation of PMMA is expected to influence the morphology and mechanical behavior of the PAN-based gel polymer electrolytes. Therefore, the optimized PPM8:2 electrolyte was first examined by digital photography and direct flame exposure to evaluate its macroscopic integrity and combustion behavior. Meanwhile, the surface and cross-sectional morphologies and tensile properties of the PAN/PMMA-based electrolytes with different compositions were systematically compared to elucidate the effect of PMMA content on their structural and mechanical characteristics.

**Figure 2:**
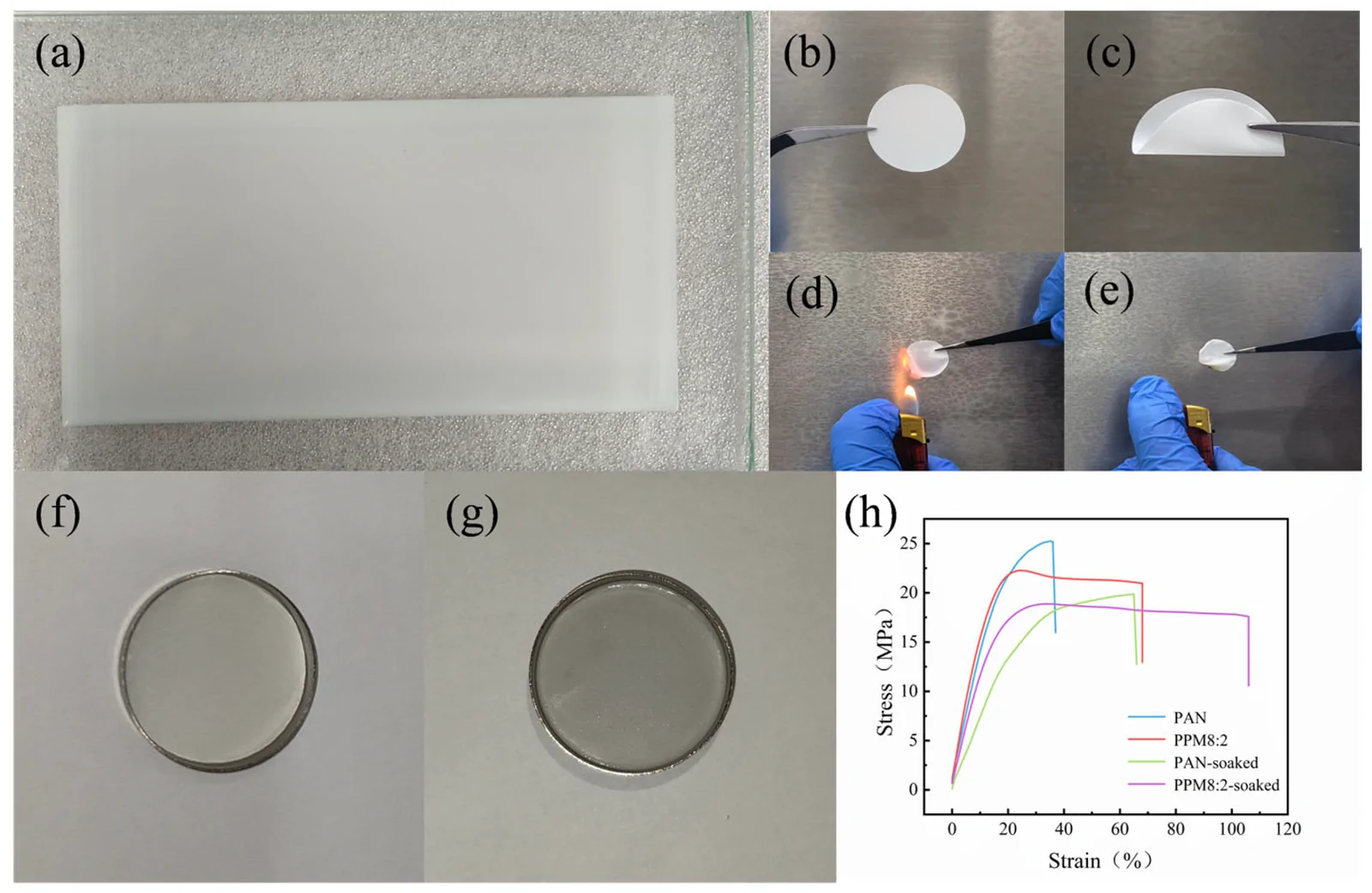
(**a**) Optical photograph of the PPM8:2 gel polymer electrolyte; (**b**–**e**) Front view, folded state, ignition behavior, and morphology after flame removal of the PPM8:2 gel polymer electrolyte, respectively; (**f**,**g**) optical photographs of the PPM8:2 gel polymer electrolyte before and after electrolyte uptake, respectively; (**h**) stress–strain curves of the PAN-based and PPM8:2 electrolytes before and after electrolyte uptake.

Figure 2a–c shows the digital photographs of the fabricated PPM8:2 electrolyte membrane. The membrane exhibits a semi-transparent milky-white appearance and good environmental stability. Moreover, it can be repeatedly bent without obvious cracking or mechanical failure, demonstrating excellent flexibility and mechanical integrity. To further evaluate the combustion behavior of the optimized electrolyte membrane, a direct flame exposure test was conducted, as shown in Figure 2d,e. Upon direct exposure to an external flame, the PPM8:2 membrane exhibits limited sustained combustion and rapidly extinguishes after removal of the ignition source. This behavior indicates that the PPM8:2 electrolyte membrane possesses a certain resistance to sustained combustion. However, this qualitative flame test reflects only the combustion behavior of the electrolyte membrane itself and should not be interpreted as a comprehensive assessment of cell-level safety.

To further evaluate the electrolyte uptake and dimensional stability of the PPM8:2 membrane under electrolyte-containing conditions, its changes before and after electrolyte uptake were investigated. As shown in Figure 2f,g, the membrane retains its overall shape without obvious macroscopic deformation after electrolyte uptake. After immersion in the liquid electrolyte for 1 h, the mass of the PPM8:2 membrane increases from 20.35 to 28.65 mg, corresponding to an electrolyte uptake of approximately 40.8%. Meanwhile, the membrane diameter remains approximately 19 mm, while the thickness increases slightly from 0.060 to 0.065 mm, corresponding to an increase of approximately 8.3%. These results indicate that the PPM8:2 membrane can effectively accommodate the liquid electrolyte while maintaining stable in-plane dimensions, with only limited expansion in the thickness direction.

Mechanical robustness is essential for maintaining stable contact between the gel polymer electrolyte and electrodes [47]. To evaluate their mechanical stability under electrolyte-containing conditions, the PAN-based and PPM8:2 membranes were also tested after electrolyte uptake, and the corresponding samples are denoted as PAN-soaked and PPM8:2-soaked, respectively. Figure 2h presents the stress–strain curves of the PAN-based and PPM8:2 electrolytes before and after electrolyte uptake. The dry PAN-based electrolyte exhibits a tensile strength of approximately 25 MPa and an elongation at break of approximately 35%. In comparison, the dry PPM8:2 electrolyte shows an elongation at break of approximately 68% while maintaining a tensile strength of approximately 22 MPa, indicating improved flexibility after PMMA incorporation. After electrolyte uptake, both electrolytes exhibit decreased tensile strength and increased elongation at break, which can be attributed to the plasticizing effect of the absorbed liquid electrolyte. Notably, the electrolyte-soaked PPM8:2 membrane retains a tensile strength of approximately 19 MPa, while its elongation at break increases to approximately 105%. These results indicate that the PPM8:2 electrolyte maintains considerable mechanical strength while exhibiting enhanced deformation tolerance after electrolyte uptake, demonstrating good mechanical integrity under electrolyte-containing conditions.

**Figure 3:**
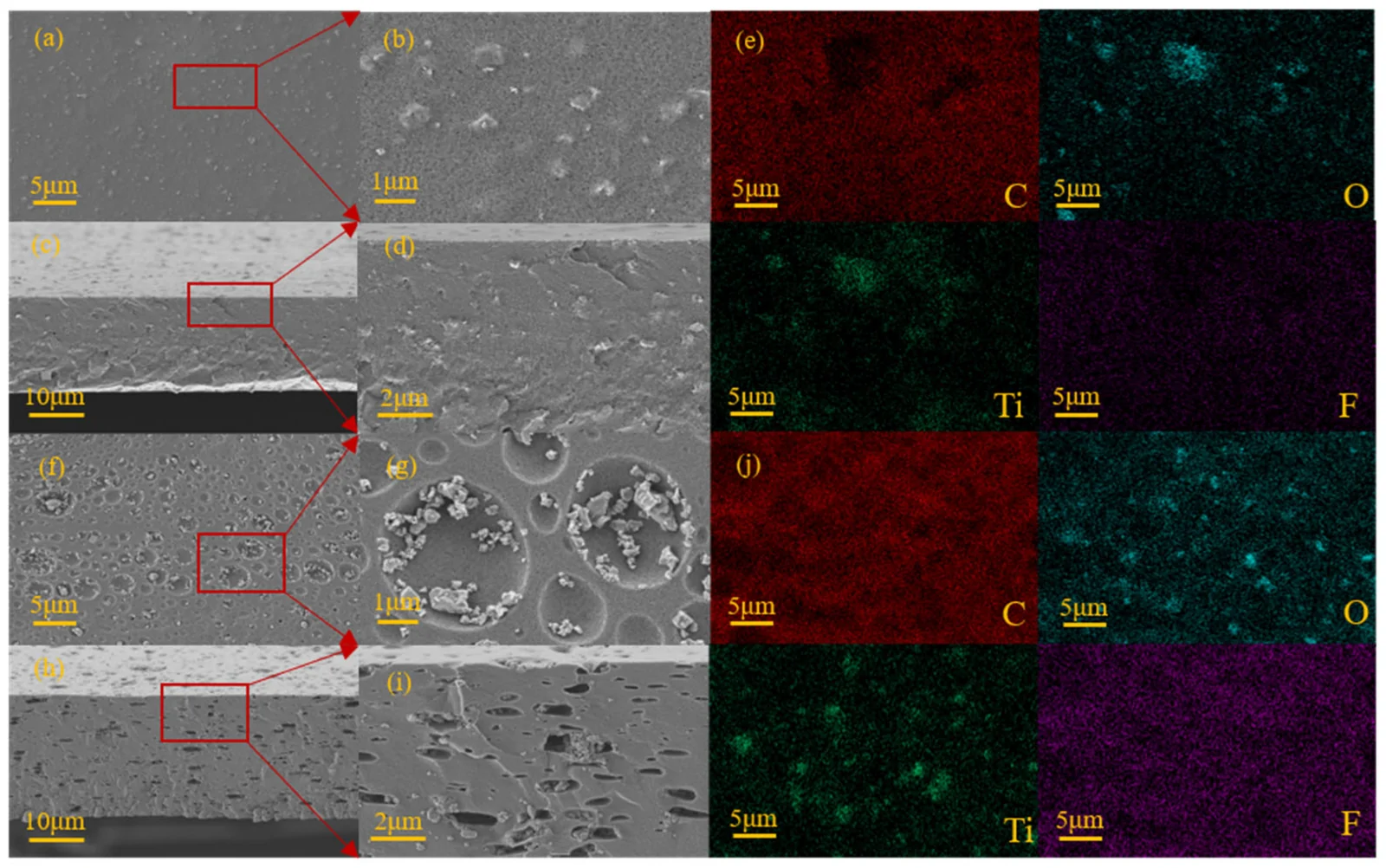
(**a**–**e**) Surface and cross-sectional morphologies and EDS elemental mappings of the PAN-based electrolyte; (**f**–**j**) surface and cross-sectional morphologies and EDS elemental mappings of the PPM8:2 gel polymer electrolyte.

Figure 3 presents the surface and cross-sectional morphologies, together with the corresponding EDS elemental mappings, of the PAN-based electrolyte (a–e) and PPM8:2 gel polymer electrolyte (f–j). The PAN-based electrolyte exhibits a relatively smooth and dense surface morphology with only a small number of nanoscale protrusions. The enlarged SEM images further reveal that LATP particles are homogeneously distributed within the PAN polymer matrix without obvious aggregation. The cross-sectional images demonstrate a continuous and compact internal structure, indicating strong interfacial contact between the polymer matrix and inorganic fillers and confirming the formation of a stable composite gel polymer network. After introducing PMMA, the PPM8:2 gel polymer electrolyte exhibits a more open microstructure. Uniformly distributed microscale pores can be observed on the surface, while LATP particles remain homogeneously embedded within the polymer matrix. The cross-sectional SEM images further reveal the formation of a continuous three-dimensional porous network. This structural evolution is attributed to the regulatory effect of PMMA on PAN chain arrangement, which suppresses the ordered packing of polymer chains and increases the free volume of the gel network. The resulting open structure facilitates electrolyte infiltration and provides continuous pathways for rapid Li^+^ migration. The EDS elemental mapping results demonstrate that C, O, Ti, and F elements are uniformly distributed throughout both PAN and PPM8:2 gel polymer electrolytes. Among them, Ti originates from the LATP ceramic filler, F is mainly associated with the LiTFSI salt, while C and O correspond to the polymer matrix. No obvious elemental aggregation is observed, indicating the homogeneous dispersion of LATP particles and LiTFSI salts within the gel polymer network. Furthermore, the incorporation of PMMA does not compromise the compositional homogeneity of the electrolyte system.

Overall, the SEM and EDS results demonstrate that an appropriate amount of PMMA can effectively regulate the microstructure of the gel polymer electrolyte while maintaining uniform dispersion of all components. The constructed open and continuous ion-transport network increases the amorphous fraction of the polymer matrix and facilitates Li^+^ migration through both polymer phases and polymer/LATP interfaces, thereby providing a favorable structural basis for enhanced ionic transport capability and stable electrochemical performance of the gel polymer electrolyte.

### 2.3. Ionic Transport and Electrochemical Stability

**Figure 4:**
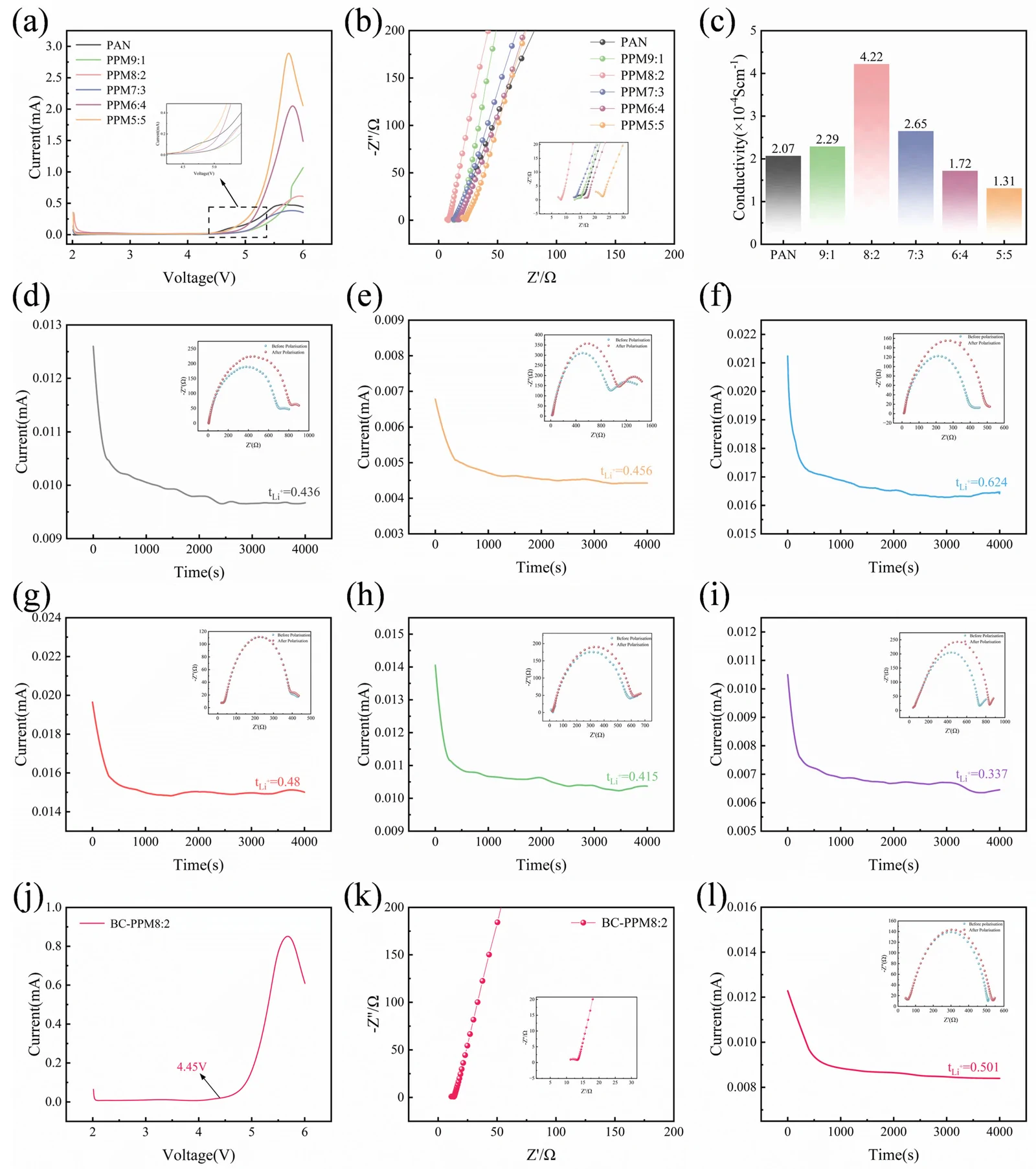
(**a**) Linear sweep voltammetry (LSV) curves of the PAN-based electrolyte and PAN/PMMA-based gel polymer electrolytes with different PAN/PMMA mass ratios; (**b**) electrochemical impedance spectroscopy (EIS) Nyquist plots of the PAN-based electrolyte and PAN/PMMA-based gel polymer electrolytes with different PAN/PMMA mass ratios; (**c**) ionic conductivities of the corresponding electrolytes; (**d**–**i**) direct-current (DC) polarization curves of Li|electrolyte|Li symmetric cells employing the PAN-based electrolyte and PAN/PMMA-based gel polymer electrolytes with different PAN/PMMA mass ratios (insets: electrochemical impedance spectra of the symmetric cells recorded before and after polarization); (**j**–**l**) Electrochemical properties of the blade-coated PPM8:2 gel polymer electrolyte (BC-PPM8:2) prepared by doctor blade coating.

Figure 4a presents the linear sweep voltammetry (LSV) curves of the PAN-based electrolyte and PAN/PMMA-based gel polymer electrolytes with different compositions. All gel polymer electrolytes exhibit negligible oxidation currents up to relatively high potentials, indicating broad electrochemical stability windows and their suitability for lithium metal battery applications. With increasing PMMA content, the oxidative stability of the gel polymer electrolytes initially improves and then decreases. Among all samples, the PPM8:2 gel polymer electrolyte exhibits the highest oxidation decomposition potential of approximately 4.75 V (vs. Li/Li^+^), which is higher than that of the PAN-based electrolyte. This improvement may be associated with the optimized gel network structure and the altered polymer environment induced by PMMA incorporation. However, excessive PMMA addition leads to a slight decrease in oxidative stability, suggesting that an appropriate PMMA content is important for achieving favorable electrochemical stability.

To further investigate the influence of PAN/PMMA composition on ionic transport behavior, electrochemical impedance spectroscopy (EIS) measurements were performed, and the corresponding Nyquist plots are shown in Figure 4b. All samples exhibit typical high-frequency semicircular features, where the intercept on the real axis corresponds to the bulk resistance of the gel polymer electrolytes. Significant differences in impedance are observed among the samples, with the PPM8:2 gel polymer electrolyte showing the lowest bulk resistance, indicating enhanced ionic transport capability.

The room-temperature ionic conductivities calculated from the EIS results are presented in Figure 4c. The PAN-based electrolyte exhibits an ionic conductivity of 2.07 × 10^−4^ S cm^−1^. With increasing PMMA content, the ionic conductivity gradually increases and reaches a maximum value of 4.22 × 10^−4^ S cm^−1^ at a PAN/PMMA mass ratio of 8:2, which is approximately twice that of the PAN-based electrolyte. Further increasing the PMMA ratio leads to a gradual decrease in ionic conductivity, with the PPM5:5 sample showing a reduced value of 1.31 × 10^−4^ S cm^−1^.

Figure 4d–i presents the chronoamperometric polarization curves and the corresponding impedance spectra of PAN and PAN/PMMA-based gel polymer electrolytes with different compositions. The related polarization currents and impedance parameters are listed in Table 1. With increasing polarization time, the current of all gel polymer electrolytes gradually decreases and eventually reaches a steady state, indicating the establishment of a steady-state Li^+^ transport process. According to the Bruce–Vincent–Evans method, the PAN-based electrolyte exhibits a lithium-ion transference number of 0.436. With the incorporation of PMMA, the *t_Li_^+^* values show an initial increase followed by a subsequent decrease. Among all samples, the PPM8:2 gel polymer electrolyte delivers the highest *t_Li_^+^* value of 0.624, which is significantly higher than that of the PAN-based electrolyte. Further increasing the PMMA content results in a gradual decrease in *t_Li_^+^*, reaching 0.337 for the PPM5:5 sample. A higher lithium-ion transference number indicates that a larger fraction of ionic current is contributed by Li^+^ migration, which can effectively suppress concentration polarization and promote more homogeneous ion transport. The enhanced *t_Li_^+^* of the PPM8:2 gel polymer electrolyte can be attributed to the structural regulation of PAN polymer chains by PMMA and the optimized Li^+^ transport environment within the gel network, which is consistent with its lower bulk resistance and higher ionic conductivity. In contrast, excessive PMMA incorporation may disrupt the continuity of effective ion transport pathways and restrict Li^+^ migration, resulting in decreased Li^+^ transport selectivity. Therefore, the optimized PPM8:2 gel polymer electrolyte achieves a favorable balance between ionic conductivity and lithium-ion transference capability, enabling efficient Li^+^ transport with reduced polarization effects. These characteristics provide a favorable electrochemical foundation for achieving improved rate capability and long-term cycling stability in lithium metal batteries.

To evaluate the influence of the fabrication process on the electrochemical properties of the gel polymer electrolyte, a PPM8:2 membrane with identical composition was prepared by doctor blade coating as a control sample (denoted as BC-PPM8:2). The electrochemical stability, ionic conductivity, and lithium-ion transport behavior of BC-PPM8:2 were compared with those of the DIW-printed PPM8:2 electrolyte. As shown in Figure 4j–l, the BC-PPM8:2 electrolyte exhibits an oxidation decomposition potential of 4.45 V, an ionic conductivity of 2.438 × 10^−4^ S cm^−1^, and a lithium-ion transference number of 0.501. Compared with the DIW-printed PPM8:2 electrolyte, the blade-coated sample shows slightly lower ionic transport capability, while maintaining a comparable electrochemical stability window and lithium-ion transport selectivity. The difference in electrochemical behavior may be related to the distinct membrane structures generated by the two fabrication processes.

### 2.4. Battery Performance and Interfacial Kinetics

**Figure 5:**
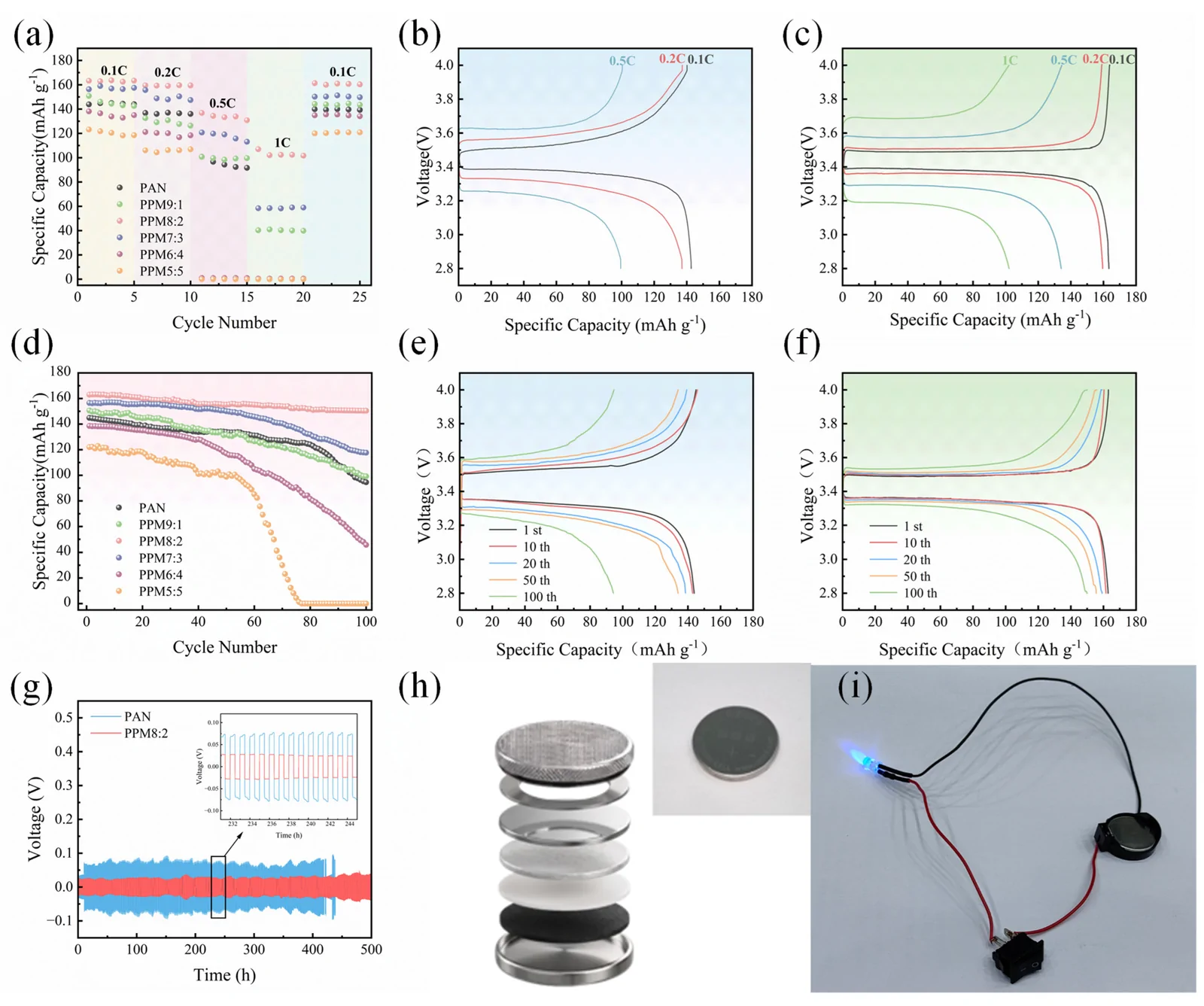
(**a**,**d**) Rate capability and long-term cycling performance of LiFePO_4_|Li cells employing the PAN-based electrolyte and PAN/PMMA-based gel polymer electrolytes with different PAN/PMMA mass ratios; (**b**,**e**) galvanostatic charge–discharge profiles of LiFePO_4_|Li cells employing the PAN-based electrolyte at different C-rates and selected cycle numbers; (**c**,**f**) galvanostatic charge–discharge profiles of LiFePO_4_|Li cells employing the PPM8:2 gel polymer electrolyte at different C-rates and selected cycle numbers; (**g**) galvanostatic Li plating/stripping performance of Li|GPE|Li symmetric cells employing the PAN-based and PPM8:2 gel polymer electrolytes at 0.1 mA cm^−2^ (inset: enlarged voltage profiles from 232 to 244 h); (**h**,**i**) LED illumination test using a CR2025 coin cell.

Figure 5a presents the rate performance of LiFePO_4_|Li cells assembled with the PAN-based electrolyte and PAN/PMMA-based gel polymer electrolytes with different compositions. As the current rate increases from 0.1 C to 1 C, all cells exhibit gradual capacity decay, which can be mainly attributed to increased polarization and limited Li^+^ transport kinetics under high-rate conditions. When the rate returns to 0.1 C, all cells recover most of their capacities, demonstrating excellent electrochemical reversibility of the gel polymer electrolyte systems. Among them, the PPM8:2 gel polymer electrolyte delivers the highest discharge capacity over the entire rate range and nearly restores its initial capacity after rate recovery, indicating superior rate adaptability. In contrast, further increasing the PMMA content results in more pronounced capacity degradation at high current rates. Particularly, the PPM5:5 electrolyte exhibits significantly reduced reversible capacities at 0.5 C and 1 C. This behavior may be associated with excessive PMMA incorporation, which alters the gel network structure and reduces the continuity of effective Li^+^ transport pathways, thereby limiting rapid ion migration during high-rate cycling. Figure 5d shows the long-term cycling performance of LiFePO_4_|Li cells employing different gel polymer electrolytes at 0.1 C. After 100 cycles, the PPM8:2-based battery maintains a discharge capacity of approximately 150 mAh g^−1^, which is significantly higher than that of the PAN-based electrolyte, demonstrating improved cycling stability. In comparison, electrolytes with higher PMMA contents exhibit accelerated capacity fading. In particular, the PPM5:5 cell experiences a rapid capacity decrease after approximately 80 cycles, suggesting that excessive PMMA is unfavorable for maintaining stable electrode/electrolyte interfacial reactions. The charge–discharge profiles of cells employing the PAN-based and PPM8:2 electrolytes at different rates and cycling stages are compared in Figure 5b,c,e,f. With increasing current density, both systems exhibit shortened voltage plateaus and increased polarization. However, the PPM8:2 cell consistently maintains flatter charge–discharge profiles and smaller voltage hysteresis throughout the measurements, indicating improved interfacial kinetics and Li^+^ transport capability. Even after 100 cycles, the PPM8:2 cell shows only limited capacity decay and voltage deviation, further confirming its stable interfacial reaction behavior and excellent electrochemical reversibility. Therefore, the optimized PAN/PMMA mass ratio of 8:2 achieves an effective balance among ionic transport capability, interfacial stability, and cycling performance, enabling the PPM8:2 gel polymer electrolyte to deliver superior overall electrochemical properties.

To further evaluate the compatibility of the gel polymer electrolytes with lithium metal, galvanostatic Li plating/stripping tests were conducted using Li|GPE|Li symmetric cells at a current density of 0.1 mA cm^−2^. As shown in Figure 5g, the PPM8:2-based symmetric cell exhibits a lower and more stable polarization voltage than the PAN-based cell during prolonged cycling. The enlarged profiles from 232 to 244 h further show that the polarization voltage of the PPM8:2 cell is approximately ±0.03 V, which is substantially lower than that of the PAN-based cell (approximately ±0.07 V). Moreover, the PPM8:2 cell maintains relatively stable Li plating/stripping behavior for approximately 500 h without a pronounced increase in polarization. These results demonstrate the improved compatibility and interfacial stability of the PPM8:2 electrolyte against lithium metal.

To further demonstrate the practical applicability of the PPM8:2 gel polymer electrolyte, CR2025 coin cells were assembled and employed for an LED illumination test. As shown in Figure 5h,i, the coin cell can stably power an LED under ambient conditions, demonstrating efficient ion transport and stable electrode/electrolyte interfacial contact. These results further demonstrate the practical feasibility of the PPM8:2 gel polymer electrolyte in lithium metal battery systems.

**Figure 6:**
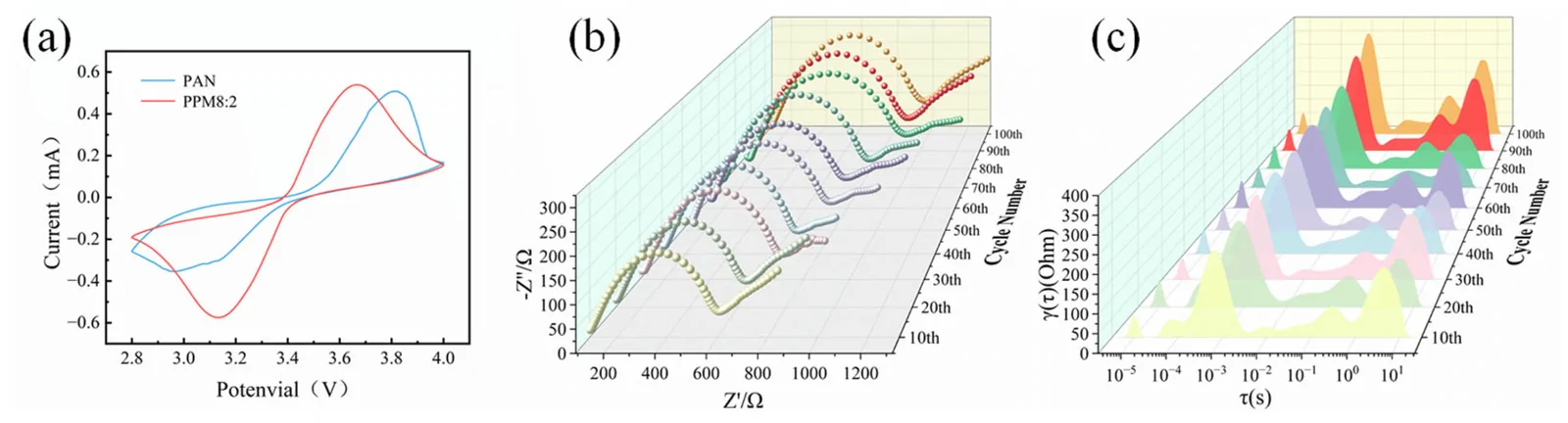
(**a**) Cyclic voltammetry (CV) curves of LiFePO_4_|Li cells employing the PAN-based electrolyte and PPM8:2 gel polymer electrolyte; (**b**) electrochemical impedance spectroscopy (EIS) spectra of LiFePO_4_|Li cells employing the PPM8:2 gel polymer electrolyte during cycling; (**c**) distribution of relaxation times (DRT) analysis of LiFePO_4_|Li cells employing the PPM8:2 gel polymer electrolyte during cycling.

Figure 6a presents the cyclic voltammetry (CV) curves of LiFePO_4_|Li cells assembled with the PAN-based and PPM8:2 gel polymer electrolytes. Both systems exhibit a pair of well-defined and reversible redox peaks, corresponding to the reversible Li^+^ insertion/extraction reactions between LiFePO_4_ and FePO_4_, indicating stable electrochemical reactions enabled by the gel polymer electrolyte systems. Compared with the PAN-based electrolyte, the PPM8:2-based cell exhibits higher redox peak currents and a smaller peak-to-peak potential separation (ΔE_p_), suggesting enhanced charge-transfer kinetics and reduced electrochemical polarization. Moreover, the sharper and more symmetric redox peaks observed for the PPM8:2 cell demonstrate faster and more homogeneous Li^+^ transport at the electrode/gel electrolyte interface, leading to improved electrochemical reversibility. The reduced ΔE_p_ indicates lower interfacial reaction resistance and enhanced electrochemical kinetics, which may be associated with the structural regulation of the PAN-based gel network by PMMA and the resulting improvement in the ionic transport environment. These effects optimize the ionic transport environment within the gel electrolyte and stabilize the electrode/electrolyte interface. Therefore, the PPM8:2 gel polymer electrolyte effectively suppresses polarization and facilitates rapid interfacial electrochemical reactions, which is consistent with its superior rate capability and long-term cycling stability.

Figure 6b presents the evolution of electrochemical impedance spectra (EIS) of the LiFePO_4_|Li battery assembled with the PPM8:2 gel polymer electrolyte at different cycling stages. With increasing cycle numbers, the Nyquist plots gradually shift toward the high impedance region, indicating a slight increase in internal resistance during prolonged cycling. Nevertheless, the impedance growth remains limited after 100 cycles, demonstrating that the PPM8:2 gel polymer electrolyte can effectively maintain a stable electrode/electrolyte interface and suppress continuous accumulation of interfacial polarization. This behavior can be attributed to the optimized gel network structure and improved interfacial adaptability, which contribute to maintaining efficient Li^+^ transport pathways. To further elucidate the evolution of electrochemical kinetics during cycling, the impedance spectra were analyzed using the distribution of relaxation times (DRT) method [48], as shown in Figure 6c. Multiple characteristic peaks are observed in different relaxation time regions, corresponding to interfacial film formation, charge-transfer processes, and Li^+^ diffusion behavior. Although the intensities of these peaks vary slightly during cycling, their positions remain almost unchanged, indicating that the dominant electrochemical processes within the battery are well preserved. The characteristic peak associated with charge-transfer kinetics in the intermediate-frequency region exhibits only a slight increase, suggesting limited growth of interfacial resistance between the electrode and gel electrolyte. Meanwhile, the low-frequency diffusion-related peak shows no obvious deterioration, confirming the sustained Li^+^ transport ability of the gel polymer electrolyte during long-term cycling. Therefore, the PPM8:2 gel polymer electrolyte effectively stabilizes the electrode/electrolyte interface and maintains efficient Li^+^ migration, providing a favorable kinetic foundation for the excellent cycling stability of the LiFePO_4_|Li battery.

## 3. Materials and Methods

### 3.1. Materials

PVDF (Mn = 600,000, Arkema, Colombes, France), poly(acrylonitrile) (PAN, Mw = 85,000, Shanghai, China), poly(methyl methacrylate) (PMMA, Mw = 35,000, Shanghai, China), Lithium bis(trifluoromethanesulfonyl)imide (LiTFSI, 99%, Macklin, Shanghai, China), Lithium aluminum titanium phosphate (LATP, average particle size ~600 nm, Shenzhen Kejing, Shenzhen, China), N,N-dimethylformamide (DMF, ≥99.9%, Aladdin, Shanghai, China), N-methyl-2-pyrrolidone (NMP, ≥99.9%), Super-P (≥99.5%), and LiFePO4 (LFP, ≥99.5%) were all obtained from Krud, Shanghai, China.

### 3.2. Preparation of Gel Polymer Electrolytes (GPEs)

Ink preparation. A certain amount of polymers was weighed according to PAN/PMMA mass ratios of 10:0, 9:1, 8:2, 7:3, 6:4, and 5:5, and dissolved in DMF under continuous magnetic stirring at 45 °C. After complete dissolution, LiTFSI (30 wt% relative to the total polymer content) was subsequently added, followed by the addition of LATP (10 wt% relative to the total polymer content), and the mixture was continuously stirred for 6 h. Subsequently, the ink was ultrasonically degassed for 30 min to remove trapped air bubbles. The electrolyte without PMMA was used as the control sample. To obtain well-dispersed printable inks, PAN, PMMA, and LATP powders were thoroughly ground using an agate mortar prior to dissolution in DMF to improve dispersion uniformity.

**Figure 7:**
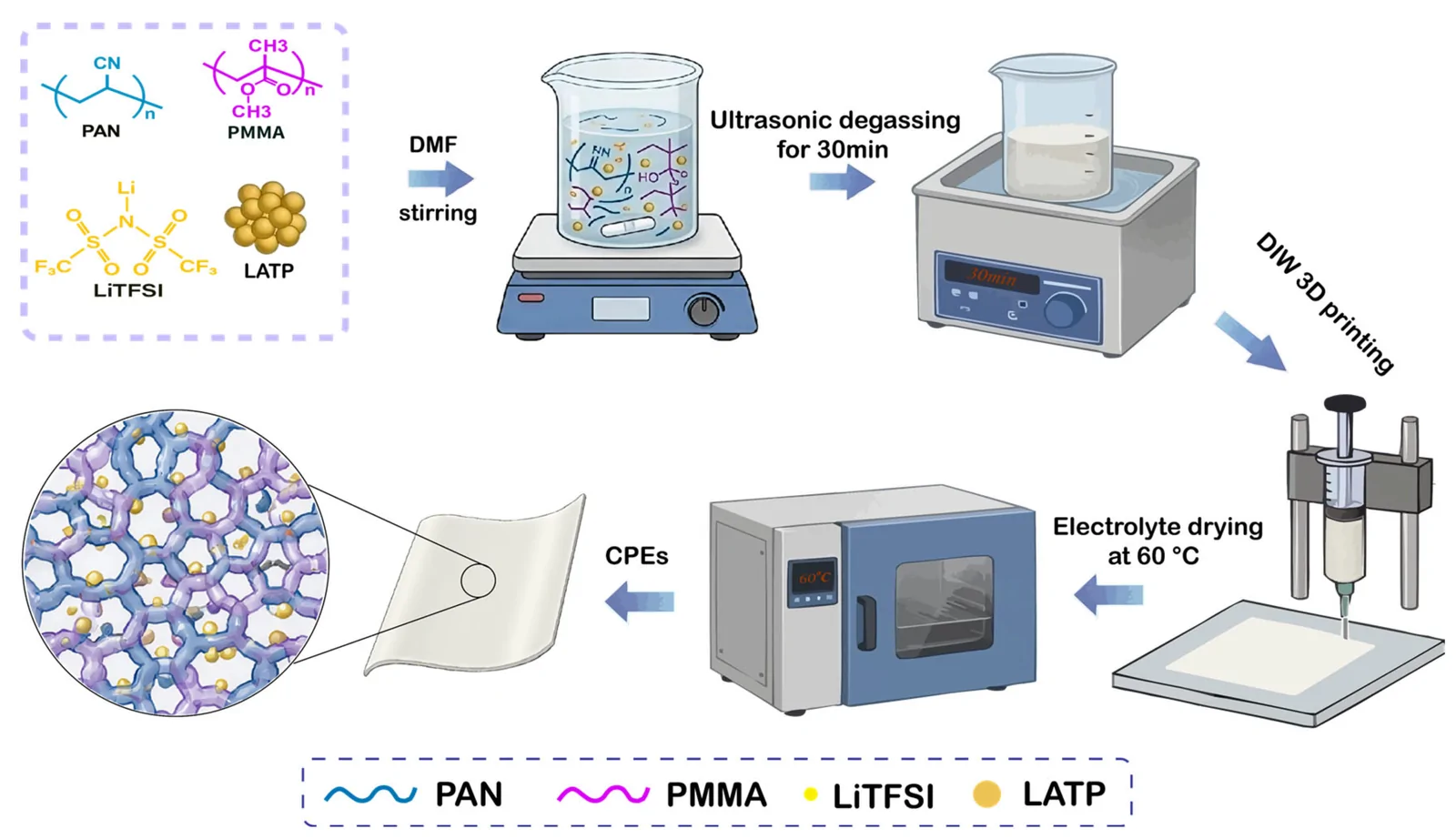
Preparation of gel polymer electrolytes.

Preparation of GPEs. The preparation process of the gel polymer electrolytes is illustrated in Figure 7. After ink preparation, the inks were loaded into syringes equipped with extrusion nozzles (inner diameter: 0.34 mm) and printed onto quartz glass substrates using a pneumatic extrusion-based 3D printer through the direct ink writing (DIW) process. Based on previously reported DIW processing conditions [49], the electrolyte membranes were fabricated using a printing speed of 40 mm s^−1^ and an infill density of 70%. During printing, the electrolyte inks were deposited layer by layer to obtain uniform composite electrolyte membranes. The printed composite electrolytes were dried in an oven for 8 h and then punched into discs with a diameter of 19 mm. The discs were subsequently transferred into an argon-filled glovebox (H_2_O < 0.01 ppm, O_2_ < 0.01 ppm) and stored for 24 h before electrochemical testing.

Preparation of the blade-coated control electrolyte. For comparison with the DIW-printed electrolyte, a PPM8:2 membrane with identical composition was prepared by doctor blade coating and denoted as BC-PPM8:2. The same PPM8:2 electrolyte ink was uniformly coated onto a quartz glass substrate using a doctor blade, followed by drying under the same conditions as the DIW-printed membrane. The resulting membrane was subsequently punched into 19 mm discs for electrochemical characterization.

The PAN/LATP/LiTFSI electrolyte without PMMA was used as the PAN-based control electrolyte and is hereafter denoted as PAN for convenience. The PAN/PMMA/LATP/LiTFSI electrolytes with PAN/PMMA mass ratios of 9:1, 8:2, 7:3, 6:4, and 5:5 were denoted as PPM9:1, PPM8:2, PPM7:3, PPM6:4, and PPM5:5, respectively.

### 3.3. Cathode Preparation and Cell Assembly

LiFePO_4_ (LFP) cathodes were prepared using commercial LFP as the active material, Super-P as the conductive additive, and poly(vinylidene fluoride) (PVDF) as the binder. LFP, Super-P, and PVDF were mixed at a mass ratio of 8:1:1, followed by the addition of N-methyl-2-pyrrolidone (NMP) to obtain a homogeneous slurry. The resulting slurry was uniformly coated onto aluminum foil and vacuum-dried at 60 °C for 36 h to remove residual NMP. The dried electrodes were punched into circular discs with a diameter of 14 mm. The mass of LFP active material in each cathode was approximately 2–3 mg, corresponding to an areal loading of approximately 1.3–2.0 mg cm^−2^. PVDF served as the binder to maintain the mechanical integrity of the cathode composite and ensure sufficient adhesion to the aluminum current collector [50]. The specific capacities were calculated based on the actual mass of LFP active material.

CR2025 coin cells were assembled using LiFePO_4_ (LFP) as the cathode and lithium metal as the anode. Prior to assembly, all cell components were vacuum-dried at 60 °C for 12 h. To reduce interfacial resistance and facilitate Li^+^ transport across the electrode/electrolyte interface, approximately 10 μL of liquid electrolyte (1 M LiPF_6_ in DMC:EC:EMC = 1:1:1 Vol% with 1.5% LiDFOB) was introduced onto the electrolyte membrane during cell assembly. The assembled cells were aged for 12 h before electrochemical testing.

### 3.4. Materials Characterization

The crystal structures of the dried GPE membranes and their raw materials (PMMA, PAN, and LATP) were characterized using an X-ray diffractometer (XRD, DX-2700, Dandong, China) with Cu-Kα radiation operated at 40 kV and 30 mA. The diffraction patterns were collected over a 2θ range of 10–90° with a scanning rate of 0.10° min^−1^. The surface and cross-sectional morphologies of the GPE membranes were characterized using scanning electron microscopy (SEM, Phenom Spectra G2, Shanghai, China). The cross-sectional samples were obtained by fracturing the GPE membranes in liquid nitrogen, and all samples were sputter-coated with gold prior to SEM observation. Elemental mapping was performed using energy-dispersive X-ray spectroscopy (EDS, Phenom Spectra G2, Shanghai, China) to evaluate the dispersion of ceramic fillers within the composite electrolyte. The tensile properties of the GPE membranes, which were cut into rectangular specimens, were measured using a universal testing machine (WDW-5, Tenson, Jinan, China). To evaluate the mechanical properties under electrolyte-containing conditions, the PAN-based and PPM8:2 membranes were immersed in the liquid electrolyte for 1 h. After immersion, the membranes were removed, and excess electrolyte on the surface was gently wiped off before tensile testing. The thermal properties of the electrolytes were investigated using thermogravimetric analysis (TGA, TG 209 F3 Tarsus, NETZSCH, Selb, Germany) and differential scanning calorimetry (DSC, DSC 3+, METTLER TOLEDO, Greifensee, Switzerland). All measurements were carried out under a nitrogen atmosphere over a temperature range of 30–800 °C at a heating rate of 10 °C min^−1^. The functional groups on the surface of the cut GPE membranes were characterized by Fourier transform infrared spectroscopy (FTIR, Spectrum 100, PerkinElmer, Waltham, MA, USA) in the wavenumber range of 500–4000 cm^−1^.

### 3.5. Electrochemical Measurements

Electrochemical impedance spectroscopy (EIS) measurements were performed using a symmetric stainless steel/electrolyte/stainless steel (SS/GPE/SS) cell configuration at room temperature. The ionic conductivity of the electrolyte membranes was calculated according to Equation (1):

(1) $$\sigma =\frac{L}{R\;S}$$

where *σ* is the ionic conductivity (S cm^−1^); *L* is the thickness of the electrolyte membrane (cm); *R* is the bulk resistance of the membrane obtained from the impedance spectra (Ω); and *S* is the contact area between the electrolyte membrane and the stainless steel electrodes (cm^2^).

Linear sweep voltammetry (LSV) measurements were performed using a lithium/electrolyte/stainless steel (Li/GPE/SS) cell configuration. The voltage was scanned from 2 to 6 V at a scan rate of 0.005 V s^−1^ to evaluate the electrochemical stability window of the electrolyte.

The lithium-ion transference number (*t_Li_^+^*) was determined using a symmetric lithium/electrolyte/lithium (Li/GPE/Li) cell configuration. Electrochemical impedance spectra were recorded before and after DC polarization, and the lithium-ion transference number was calculated according to Equation (2):

(2) $$t^{+}=\frac{I_{S}(\Delta V-R_{0}I_{0})}{I_{0}(\Delta V-R_{S}I_{S})}$$

where t_Li+_ is the lithium-ion transference number, *I*_0_ and *Is* are the initial current and the steady-state current after 4000 s of polarization, respectively, *R*_0_ and *Rs* represent the initial interfacial resistance and the steady-state interfacial resistance after 4000 s, respectively, and Δ*V* is the applied polarization voltage of 0.01 V.

The interfacial stability of the gel polymer electrolytes against lithium metal was further evaluated by galvanostatic Li plating/stripping tests using Li|GPE|Li symmetric cells at room temperature. The symmetric cells were cycled at a current density of 0.1 mA cm^−2^.

Cyclic voltammetry (CV) was employed to evaluate the redox behavior and electrochemical reversibility of the assembled cells. The measurements were carried out on a DH7000 electrochemical workstation within a voltage window of 2.8–4.0 V at a scan rate of 0.2 mV s^−1^. The electrochemical kinetics and reversibility were evaluated based on the positions of the oxidation/reduction peaks and the potential separation between the corresponding redox couples.

## 4. Conclusions

In this work, a series of PAN/PMMA/LATP gel polymer electrolytes were successfully fabricated via direct ink writing (DIW) 3D printing technology. The effects of PAN/PMMA composition on the microstructure, thermal stability, mechanical properties, ionic transport behavior, and electrochemical performance of the gel polymer electrolytes were systematically investigated. The results demonstrate that the incorporation of PMMA effectively modifies the arrangement of PAN polymer chains, reduces the crystallinity of the polymer matrix, increases the amorphous fraction, and alters the local functional-group environment of the gel network. These structural changes are accompanied by improved ionic transport properties. In addition, the uniformly dispersed LATP ceramic fillers not only reinforce the mechanical stability of the gel electrolyte but also provide additional Li^+^ transport pathways, further promoting ion migration. Among all samples, the PPM8:2 gel polymer electrolyte with a PAN/PMMA mass ratio of 8:2 exhibits the most favorable overall performance, delivering a room-temperature ionic conductivity of 4.22 × 10^−4^ S cm^−1^, a lithium-ion transference number of 0.624, and an electrochemical stability window of approximately 4.75 V (vs. Li/Li^+^). Furthermore, the PPM8:2 gel polymer electrolyte combines good flexibility, thermal stability, and mechanical integrity, enabling LiFePO_4_|Li cells to achieve outstanding rate capability and cycling stability. The LED illumination test using a CR2025 coin cell further confirms its stable ion transport ability and practical electrochemical reliability. Overall, the combination of PAN/PMMA compositional regulation, LATP ceramic reinforcement, and DIW-based fabrication provides an effective strategy for constructing gel polymer electrolytes with favorable structural characteristics and electrochemical performance. This work provides an effective strategy for designing and fabricating printable high-performance gel polymer electrolytes and offers new insights into the development of advanced lithium metal batteries.

## Figures and Tables

**Table 1:** Measured parameters and calculated lithium-ion transference numbers (*t_Li_^+^*) of gel polymer electrolytes at room temperature.

GPEs	*I*_0_/mA	*Is*/mA	*R*_0_/Ω	*Rs*/Ω	*t_Li_^+^*
PAN	0.0126	0.00967	699.096	817.266	0.436
PPM9:1	0.00678	0.00443	930.131	1062.304	0.456
PPM8:2	0.02124	0.01646	396.937	489.139	0.624
PPM7:3	0.01965	0.01501	365.209	366.925	0.48
PPM6:4	0.01405	0.01037	558.446	594.741	0.415
PPM5:5	0.0105	0.00645	698.698	797.892	0.337

## Data Availability

Data are contained within the article.
